# Telomerase reverse transcriptase promotes the proliferation of human laryngeal carcinoma cells through activation of the activator protein 1

**DOI:** 10.3892/ol.2013.1344

**Published:** 2013-05-08

**Authors:** YANG JIANG, CHEN CHEN, SHI-MING CHEN, YA-QIU WANG, YONG XU, YAN WANG, ZHE CHEN, BO-KUI XIAO, ZE-ZHANG TAO

**Affiliations:** Department of Otolaryngology-Head and Neck Surgery, Renmin Hospital of Wuhan University, Wuhan 430060, P.R. China

**Keywords:** telomerase reverse transcriptase, activator protein 1, p38, extracellular regulated protein kinase, laryngeal carcinoma

## Abstract

TERT is the main functional unit of telomerase, which maintains telomere length and chromosome structure stability. TERT has been shown to act as a key factor in various biological processes, such as cell proliferation, via uncharacterized mechanisms. We transfected HEp-2 laryngeal carcinoma cells with a TERT overexpressing adenovirus (Ad-TERT) and TERT shRNA silencing adenovirus (Ad-sh-TERT), and examined the effect on TERT and the AP-1 transcription factor subunits c-Fos and c-Jun using RT-PCR and western blot analysis. *TERT* mRNA expression was quantified using RT-PCR in 24 human laryngeal carcinoma samples, and TERT protein co-expression with AP-1 was investigated in a human laryngeal carcinoma tissue microarray using quantum-dot based immunofluorescence. The effect of specific ERK and p38 inhibitors on ERK, p38, c-Jun and c-Fos phosphorylation was investigated in TERT-overexpressing HEp-2 cells. TERT overexpression led to increased TERT, c-Jun and c-Fos mRNA and protein expression and increased cell proliferation, while TERT silencing had the opposite effects. *TERT* mRNA expression levels were positively correlated with *c-Fos* and *c-Jun* mRNA in human laryngeal carcinoma tissue. TERT and AP-1 protein were expressed at high levels and positively correlated in laryngeal carcinoma tissues. Treatment of TERT-overexpressing HEp-2 cells with specific p38 and ERK inhibitors indicated that TERT modulates the expression and phosphorylation of the AP-1 subunits c-Jun and c-Fos through the p38 and ERK signaling pathways. In conclusion, the results of this study indicate that TERT is capable of promoting cell proliferation via activation of the AP-1 subunits, c-Jun and c-Fos, in laryngeal carcinoma cells.

## Introduction

Telomeres are composed of telomeric DNA and several binding proteins, which act together as a protective cap on the ends of chromosomes (1). Healthy human somatic cells do not express telomerase, and therefore telomere size decreases with each cell division. Telomere shortening serves as a checkpoint for the initiation of cell cycle arrest, which leads to cellular senescence or aging, and apoptosis or cell death (2). Normal cells have a finite capacity for replication; however, telomerase activity is observed in more than 90% of samples from a wide range of different types of cancer.

Telomerase is a ribonucleoprotein complex whose main function is to add six nucleotide repeats onto the ends of chromosomes, in a mechanism that is dependent on its reverse transcriptase activity (TERT) and intrinsic RNA template (TERC), as well as the associated proteins, dyskerin, NOP10, NHP2 and GAR1. TERT is the major catalytic component of telomerase (3), and has been extensively studied, with several of its functional domains being mapped already. The ectopic expression of TERT is sufficient to restore telomerase activity in telomerase-negative cells and increase cell division in a number of cell types (4). Downregulation of TERT in telomerase-positive cancer cells results in growth arrest. These findings demonstrate that TERT or telomerase activity is required for cancer cell immortalization and proliferation (5,6).

TERT and telomerase have other biological functions beyond telomere lengthening, including the protection of mitochondrial function under oxidative stress conditions, promoting stem cell proliferation and enhancing DNA repair (7). Recently, several additional activities for TERT have been reported, which indicates that TERT is able to exert telomere-independent biological functions, including promoting cell proliferation (5,8), extending cell life (6,9), delaying cell aging (10,11) and modulating cell differentiation (12). Some of these new functions do not depend on the reverse transcriptase activity of TERT (7,13).

Activating protein 1 (AP-1) is a dimeric transcription factor composed of proteins from several families containing a basic leucine zipper (bZIP) domain, which is essential for dimerization and DNA binding. The Jun (c-Jun, JunB and JunD) and Fos (c-Fos, FosB, Fra1 and Fra2) subfamilies are the major AP-1 proteins. AP-1 regulates a number of cell processes, including proliferation, inflammation, differentiation and apoptosis by contributing to both basal- and stimulus-activated gene expression. External stimuli, such as growth factors, neurotransmitters, polypeptide hormones, bacterial and viral infections, as well as a variety of physical and chemical stresses, activate AP-1 via the mitogen-activated protein kinase cascades to induce both short- and long-term gene expression changes (14,15).

TERT and telomerase are overexpressed in 85–90% of human cancers, and are closely correlated with the development of laryngeal carcinoma and the proliferation of laryngeal carcinoma cells. Using siRNA targeting, TERT is capable of inhibiting laryngeal carcinoma cell proliferation, but the mechanisms are not well understood (16). Certain investigators have reported that TERT may induce the expression of growth-related proteins, such as epidermal growth factor receptor (EGFR) in human glioma cancer cells (17), and can also interfere with the TGF-β growth factor network (18). In this study, we investigated the correlation between TERT and the major AP-1 proteins (c-Jun and c-Fos) during TERT-promoted laryngeal carcinoma cell proliferation.

## Materials and methods

### Cell lines and reagents

The human laryngeal carcinoma cell line, HEp-2, was constructed in our laboratory and stored in liquid nitrogen. Fetal bovine serum (FBS) was obtained from HyClone (Logan, UT, USA). RPMI-1640 media and 0.25% trypsin solution were purchased from Invitrogen (Carlsbad, CA, USA). The TERT, c-Fos, c-Jun and GAPDH PCR primers were synthesized by Invitrogen. The TERT antibody was purchased from Abcam (Cambridge, UK), the AP-1 antibody was obtained from Sigma-Aldrich (St. Louis, MO, USA), and the c-Fos, p-c-Fos, c-Jun, p-c-Jun, p-p38, p38, ERK, p-ERK and GAPDH antibodies were purchased from Cell Signaling Technology (Beverly, MA, USA). The cell counting kit-8 was obtained from Dongji (Kumamaoto, Japan), the quantum dots immunofluorescence detection kit was purchased from Jiayuan Quantum Dots (Wuhan, China), and the adenovirus packaging system, the negative control adenovirus Ad-HK and TERT sh-RNA cDNA were obtained from Genesil (Wuhan, China). The p38 MAPK inhibitor, SB202190, and the MEK1 and MEK2 inhibitor, U0126, were purchased from Sigma-Aldrich.

### Cell culture

The HEp-2 cell line was cultured in RMPI-1640 supplemented with 10% (FBS), 20 *μ*g/ml ampicillin and 20 *μ*g/ml kanamycin, and maintained in an incubator with 5% CO_2_ at 37°C.

### Human laryngeal carcinoma tissue samples

The human laryngeal carcinoma tissue samples were obtained from 24 laryngeal carcinoma cancer patients undergoing total laryngectomy or partial laryngectomy, and the diagnosis of laryngeal carcinoma was confirmed by pathological examination. The specimens were transfected to liquid nitrogen within 15 min of excision, and were stored at −80°C. Paraffin blocks created from these patients were used for the tissue microarray construction. This study was approved by the ethics review committee of Renmin Hospital of Wuhan University, China. Informed consent was obtained from all patients.

### Construction of TERT shRNA and overexpressing adenovirus vectors

Based on the design principles for shRNA construction, we selected RNAi target sites within the open reading frames of human *TERT*. A combination of computer algorithms and experimental validation were employed to determine the optimal siRNA sequences complementary to the target mRNA while inducing minimal immune responses. The specific base sequence of the target site of *TERT* was 5′-GTTCCTGCACTGGCTGATG-3′. The full length human *TERT* sequence was obtained from The National Center for Biotechnology Information (GenBank ID: 7015) and synthesized by Genechem (Shanghai, China).

We constructed Ad-sh-TERT, a recombinant adenovirus expressing the human TERT shRNA under the control of the immediate early cytomegalovirus promoter, and Ad-TERT, a recombinant adenovirus expressing the full length human *TERT* mRNA under the immediate early cytomegalovirus promoter. The *TERT* shRNA and the *TERT* full-length cDNA were subcloned into the *Hind*III and *Bam*HI restriction sites of the shuttle vector pGenesil-1 (Genesil) using an adenoviral vector system. The pGenesil-1 vector was homologously recombined with the pAd/PL-DEST vector in electro-competent DH5a bacteria and selected on LB plates containing ampicillin and chloramphenicol. The complete Ad-sh-TERT and Ad-TERT viruses were recovered by transfection of 10 mg *Pac*I digested DNA into human embryonic kidney (HEK 293) cells using Lipofectamine (Invitrogen) (19).

### Tissue microarray construction

Tissue microarrays (TMAs) were constructed by standard procedures using a tissue microarrayer (Beecher Instruments, Silver Spring, MD, USA) in collaboration with Guilin Fanpu Biotech Co. Ltd. (Guilin, China). Briefly, all specimens were reviewed by hematoxylin and eosin (H&E) staining and representative areas were marked in the formalin-fixed, paraffin-embedded blocks. Two cores from different invasive areas were removed from each specimen using 2-mm punch cores along the greatest dimension of each block. The cores were deposited into the recipient paraffin blocks in one of 70 cylinders. Two duplicates of each 2-mm diameter cylinder were included for each case to ensure reproducibility and homogeneous staining of the slides. Consecutive sections (4 *μ*m) of the resulting TMA paraffin blocks were sectioned to create the TMA slides.

### Cell proliferation

HEp-2 cells were seeded at 1×10^3^/well in 96-well plates in RPMI-1640 medium supplemented with 10% FBS. Twenty-four hours later, the cells were transfected with Ad-sh-TERT, Ad-TERT or Ad-HK, and proliferation was determined at 0, 24, 48 and 72 h post-transfection using the Cell Counting Kit-8, according to the manufacturer’s instructions.

### RNA extraction and RT-PCR

Total RNA was extracted using the TRIzol total RNA extraction kit (Invitrogen) according to the manufacturer’s instructions, and cDNA was prepared from 1 *μ*g total RNA using *Taq* DNA polymerase (Thermo-Fisher) and oligo (dT) primers (Thermo-Fisher). The primer sets used were *TERT* (forward: 5′-GGAGCAAGTTGCAAAGCA TTG-3′; reverse: 5′-TCCCACGACGTAGTCCATGTT-3′) to amplify a 182-bp product, *c-Fos* (forward: 5′-TGCCTCTCC TCAATGACCCTGA-3′; reverse: 5′-ATAGGTCCATGTCTG GCACGGA-3′) to amplify a 162-bp product, *c-Jun* (forward: 5′-CTCCAAGTGCCGAAAAAGGAAG-3′; reverse: 5′-CAC CTGTTCCCTGAGCATGTTG-3′) to amplify a 118-bp product and *GAPDH* (forward: 5′-CCTGTTCGACAGTCA GCCG-3′; reverse: 5′-CGACCAAATCCGTTGACTCC-3′) to amplify a 101-bp product.

The PCR conditions consisted of an initial denaturation at 95°C for 5 min; 30 cycles of 94°C for 30 sec, 60°C for 30 sec and 72°C for 1 min, followed by a final extension at 72°C for 10 min. The PCR products were separated on a 1.5% agarose gel, and visualized and photographed using a gel documentation system.

### Western blotting

HEp-2 cells were collected and lysed in buffer containing 1% Nonidet-P40 supplemented with complete protease inhibitor cocktail (Roche, Basel, Switzerland) and 2 mM dithiothreitol. The lysates were resolved using 12% SDS-PAGE, transferred to nitrocellulose membranes and immunoblotted with primary antibodies against hTERT, c-Jun, c-Fos, p-c-Jun, p-c-Fos, p-ERK, ERK, p-p38, p38 and GAPDH. Following incubation with secondary antibodies, the protein bands were detected using enhanced chemiluminescence (ECL) reagent (Thermo-Fisher). The ERK and p38 inhibitors were used at a concentration of 20 and 2 *μ*M, respectively, and cells were treated with the inhibitors 24 h prior to transfection with the Ad-TERT or AD-sh-TERT.

### Quantum-dots-based immunofluorescence

TERT and AP-1 double immunofluorescence staining using 605-QD-SA and 545-QD-SA probes was performed on the head and neck cancer tissue microarray. The TMA was deparaffinized, antigen retrieval was performed, blocked with 3% BSA and incubated with primary mouse anti-human TERT monoclonal antibody. Samples were then incubated with rabbit anti-human AP-1 monoclonal antibody, washed and incubated with biotinylated goat anti-rabbit IgG. The slides were washed and blocked, incubated with 605-QD-SA or 545-QDSA, washed and blocked, then incubated with biotinylated goat anti-mouse IgG, washed, blocked and incubated with 545-QD-SA or 605-QD-SA, and mounted and observed by fluorescence microscopy (20). The results were captured and analyzed using Nuance 2.10 (CRi, Woburn, MA, USA).

### Statistical analysis

Values were shown as the means ± SD. One-way ANOVA and Pearson’s correlation analysis were performed using SPSS (SPSS Inc., Chicago, IL, USA). P<0.05 was considered to indicate a statistically significant difference.

## Results

### TERT, c-Fos and c-Jun are overexpressed in laryngeal carcinoma cells and tissue samples

**Figure 1.**
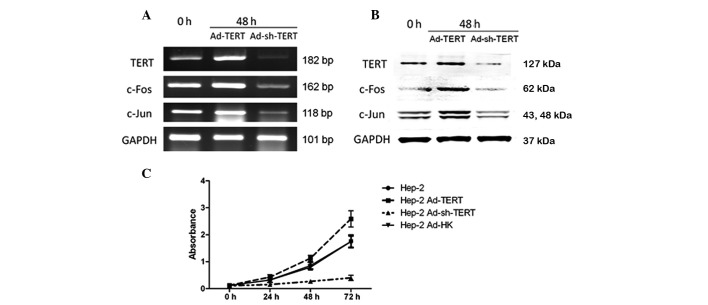
Effect of TERT overexpression (Ad-TERT) and silencing (Ad-sh-TERT) on TERT, c-Fos and c-Jun mRNA and protein expression in HEp-2 laryngeal carcinoma cells. (A) RT-PCR analysis of *TERT*, *c-Fos* and *c-Jun* mRNA expression in control, Ad-TERT and Ad-sh-TERT transfected HEp-2 cells. (B) Western blot analysis of TERT, c-Fos and c-Jun protein expression in control, Ad-TERT and Ad-sh-TERT transfected HEp-2 cells. (C) Growth curves of control, plasmid control, Ad-TERT and Ad-sh-TERT transfected HEp-2 cells; Hep-2-Ad-HK, blank control of Hep-2 Ad-TERT and Hep-2 Ad-sh-TERT.

**Figure 2.**
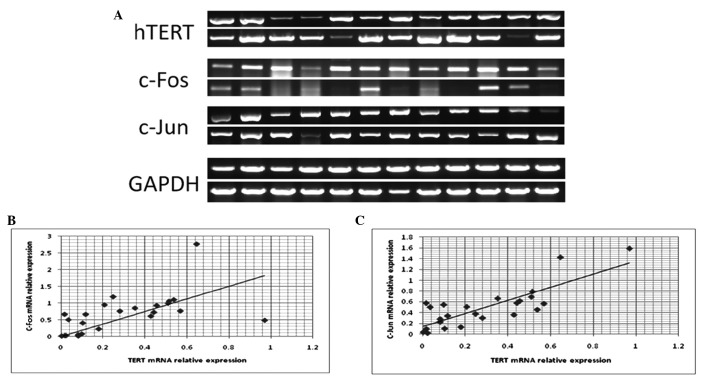
Correlation between *TERT*, *c-Fos* and *c-Jun* mRNA expression in human laryngeal carcinoma tissue samples. (A) RT-PCR analysis of *TERT*, *c-Fos* and *c-Jun* mRNA expression in 24 human laryngeal carcinoma tissue samples. A positive correlation was observed between (B) *TERT* and *c-Fos* mRNA expression levels (R^2^=0.574, P<0.01) and (C) *TERT* and *c-Jun* mRNA expression levels (R^2^=0.809, P<0.01).

**Figure 3.**
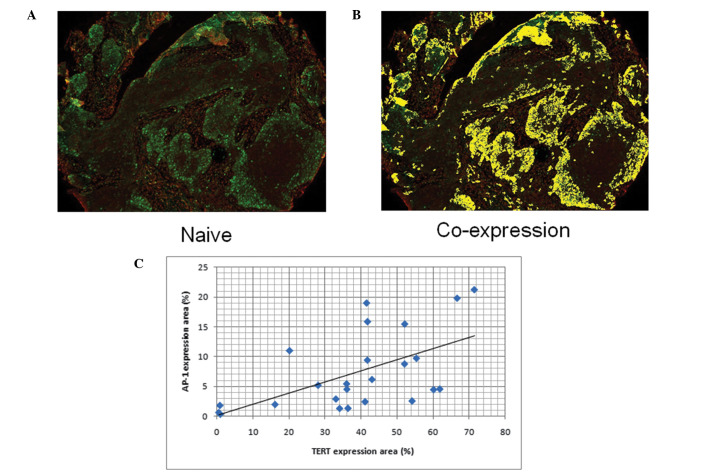
TERT and AP-1 protein expression in human laryngeal carcinoma. (A) Representative image of quantum-dot based immunofluorescence in a tissue microarray indicating that TERT (red) and AP-1 (green) are co-expressed in human laryngeal carcinoma. (B) The area of TERT and AP-1 co-expression (yellow) in human laryngeal carcinoma tissue is shown. (C) A positive correlation was observed between TERT and AP-1 expression in human laryngeal carcinoma tissue microarrays (R^2^=0.606, P<0.01).

To test the hypothesis that TERT and AP-1 are overexpressed in laryngeal carcinoma cells and therefore could contribute to laryngeal carcinoma cell proliferation, we analyzed the mRNA and protein expression levels of TERT, c-Fos and c-Jun in HEp-2 cells and human laryngeal carcinoma tissue samples. TERT, c-Fos and c-Jun mRNA and protein were all observed to be expressed at high levels in HEp-2 cells (Fig. 1A and B) and laryngeal carcinoma tissue samples (Figs. 2A, 3A and B).

### TERT modulates the proliferation of HEp-2 cells

When TERT expression was suppressed by the transfection of Ad-sh-TERT, HEp-2 cell proliferation was inhibited in a time-dependent manner (P<0.01). However, when TERT was overexpressed by the transfection of Ad-TERT, the proliferation of HEp-2 cells increased at 72 h compared with the negative control Ad-HK transfected cells (Fig. 1C, P<0.05).

### TERT modulates the expression of c-Fos and c-Jun

Following treatment with Ad-TERT and Ad-sh-TERT for 48 h, the mRNA and protein levels of TERT, c-Fos and c-Jun changed significantly in HEp-2 cells (Fig. 1A and B). Transfection of Ad-TERT led to an increase in TERT, c-Fos and c-Jun mRNA and protein expression. The transfection of Ad-sh-TERT led to reduced TERT, c-Fos and c-Jun mRNA and protein expression.

### TERT is co-expressed with AP-1

To examine the correlation between *TERT* and the AP-1 subunits *c-Fos* and *c-Jun*, we analyzed the correlation between *TERT*, *c-Fos* and *c-Jun* mRNA expression in 24 laryngeal carcinoma tissue samples using RT-PCR (Fig. 2A). The correlation coefficient between *TERT* and *c-Fos* mRNA expression in laryngeal carcinoma tissue samples was 0.574 (Fig. 2B; P<0.01), and the correlation coefficient between *TERT* and *c-Jun* mRNA expression was 0.809 (Fig. 2C; P<0.01). These data indicate that *TERT* expression is significantly and positively correlated with both *c-Fos* and *c-Jun* expression in laryngeal carcinoma.

We also determined the correlation between the protein expression levels of TERT and AP-1 in a laryngeal carcinoma tissue microarray using quantum-dot based immunofluorescence (Fig. 3A and B). A significant positive correlation was observed between TERT and AP-1 expression in laryngeal carcinoma cells (Fig. 3C, R^2^= 0.606; P<0.01).

### TERT modulates the p38/ERK signaling pathway

**Figure 4.**
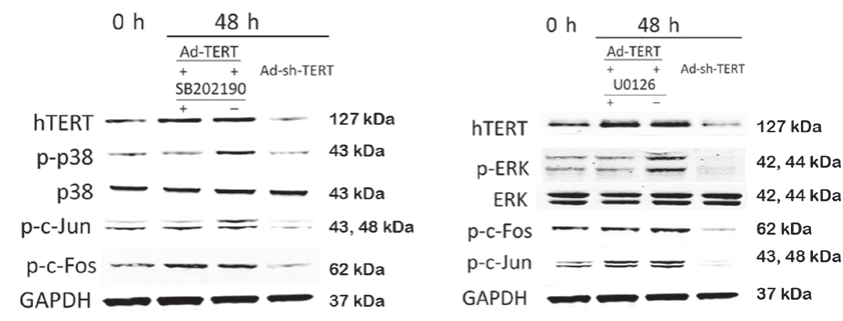
Effect of TERT overexpression with modulation of the p38 and ERK signaling pathways on c-Fos and c-Jun expression and activation in the HEp-2 laryngeal carcinoma cells. (A) Western blot analysis of the effect of TERT overexpression (Ad-sh-TERT) and silencing (Ad-Sh-TERT) on the p38 signaling pathway and c-Fos and c-Jun expression in the presence and absence of the p38 inhibitor, SB202190. (B) Western blot analysis of the effect of TERT overexpression (Ad-sh-TERT) and silencing (Ad-Sh-TERT) on the ERK signaling pathway and c-Fos and c-Jun expression in the presence and absence of the ERK inhibitor, U0126.

It has been reported that p38/ERK activation induces AP-1 expression; therefore, we examined the relationship between TERT, p38, ERK, JNK and AP-1 in HEp-2 laryngeal carcinoma cells. Compared to the control HEp-2 cells, the levels of phosphorylated p38 (p-p38), phosphorylated ERK (p-ERK), phosphorylated c-Jun (p-c-Jun) and phosphorylated c-Fos (p-c-Fos) increased following transfection with Ad-TERT (Fig. 4). Conversely, the levels of p-p38, p-ERK, p-c-Jun and p-c-Fos were reduced following transfection with Ad-sh-TERT. In the presence of SB202190, a specific inhibitor of p38, overexpression of TERT did not lead to increased p38 or c-Jun phosphorylation. In the presence of U1026, a specific inhibitor of ERK, transfection with Ad-TERT did not lead to increased levels of p-ERK; however, ERK inhibition did not prevent TERT-induced c-Jun and c-Fos phosphorylation (Fig. 4).

## Discussion

In this study, we overexpressed and silenced the expression of TERT in the human laryngeal carcinoma cell line, HEp-2, using adenovirus-based vectors. Overexpression of TERT markedly accelerated HEp-2 cell proliferation, while the silencing of TERT significantly decreased the rate of HEp-2 proliferation to approximately 30% of the levels observed in the control cells. These results indicate that *TERT* gene expression is important in cell proliferation in the HEp-2 human laryngeal carcinoma cell line. Although the ability of TERT to promote proliferation has already been proven in various cells, including fibroblasts, epithelial cells, bone marrow mesenchymal stem cells, cancer stem cells and tumor cells (4,5,8), the molecular mechanism remains unclear. The whole genome analysis by Takano *et al* (21) indicated that the expression of genes in the PI3K, Akt and Caspase pathways, which are associated with cell proliferation and apoptosis, altered significantly in cells that overexpressed TERT. Yang *et al* (4) reported that TERT is capable of promoting the proliferation of human embryonic stem cells in a mechanism that is associated with the expression of cyclin D1. This mechanism may vary between normal and tumor cells, and even between different tumor cell types.

AP-1 is important in eukaryotic cell proliferation, cell cycle regulation and resistance to apoptosis. The biological function of AP-1 is closely related to the major subunits, the transcription factors c-Jun and c-Fos, which form homologous or heterogeneous dimers (22). AP-1 regulates a number of cell processes, including proliferation, inflammation, differentiation and apoptosis (23,24). In this study, c-Fos and c-Jun expression were significantly increased in TERT-overexpressing HEp-2 cells, and decreased in TERT-silenced HEp-2 cells. Additionally, the expression of *c-Fos* and *c-Jun* mRNA were both positively correlated with *TERT* expression in human laryngeal carcinoma tissues. Park *et al* (25) reported that TERT interacts with BRG1 to activate transcription of the Wnt/β-catenin signaling pathway and the downstream target genes. This indicates that TERT may play a role in this mechanism that is similar to a transcription factor. Therefore, we may deduce that TERT acts as a transcription factor to modulate the expression of *c-Fos* and *c-Jun*, as the expression of TERT is closely correlated with c-Fos and c-Jun mRNA and protein expression in HEp-2 laryngeal carcinoma cells, and is positively correlated with *c-Fos* and *c-Jun* mRNA expression in human laryngeal carcinoma tissues.

AP-1 activation occurs at the transcriptional and post-translational levels. The predominant AP-1 activation signals are mediated via the mitogen-activated protein kinase (MAPK) cascade (26). The MAPK pathway converges on three MAP kinases, extracellular regulated kinase (ERK), Jun N-terminal kinase (JNK) and p38. AP-1 may be activated by all of the MAPK pathways (27). We demonstrated that TERT affects the expression of c-Fos and c-Jun at both the transcriptional and translational level, and c-Fos and c-Jun phosphorylation was significantly altered in TERT-overexpressing and TERT-silenced HEp-2 cells. Therefore, to identify whether TERT is capable of modulating c-Fos and c-Jun activation at a post-translational level, we investigated p38, JNK (data not shown) and ERK expression and phosphorylation. TERT overexpression promoted p38 phosphorylation and c-Jun phosphorylation. Phosphorylation of c-Jun in response to TERT overexpression was inhibited in the presence of a specific p38 inhibitor, indicating a correlation between the phosphorylation of c-Jun and p38. However, a specific ERK inhibitor did not prevent phosphorylation of c-Fos or c-Jun in TERT-overexpressing cells. It is known that p38 phosphorylation specifically leads to c-Jun phosphorylation, and that ERK phosphorylation is required for c-Fos phosphorylation (28).

In conclusion, this study indicates that TERT is important in cell proliferation in laryngeal carcinoma. TERT induces altered expression and activation of the AP-1 subunits c-Fos and c-Jun, via the MAPK pathway, which may explain the increased proliferation observed in cells that overexpress TERT.
